# Assessment of the Biological Pathways Targeted by Isocyanate Using N-Succinimidyl N-Methylcarbamate in Budding Yeast *Saccharomyces cerevisiae*

**DOI:** 10.1371/journal.pone.0092993

**Published:** 2014-03-24

**Authors:** Gajendra Kumar Azad, Vikash Singh, Raghuvir S. Tomar

**Affiliations:** Laboratory of Chromatin Biology, Department of Biological Sciences, Indian Institute of Science Education and Research, Bhopal, India; Heidelberg University, Germany

## Abstract

Isocyanates, a group of low molecular weight aromatic and aliphatic compounds possesses the functional isocyanate group. They are highly toxic in nature hence; we used N-succinimidyl N-methylcarbamate (NSNM), a surrogate chemical containing a functional isocyanate group to understand the mode of action of this class of compounds. We employed budding yeast *Saccharomyces cerevisiae* as a model organism to study the pathways targeted by NSNM. Our screening with yeast mutants revealed that it affects chromatin, DNA damage response, protein-ubiquitylation and chaperones, oxidative stress, TOR pathway and DNA repair processes. We also show that NSNM acts as an epigenetic modifier as its treatment causes reduction in global histone acetylation and formation of histone adducts. Cells treated with NSNM exhibited increase in mitochondrial membrane potential as well as intracellular ROS levels and the effects were rescued by addition of reduced glutathione to the medium. We also report that deletion of SOD1 and SOD2, the superoxide dismutase in *Saccharomyces cerevisiae* displayed hypersensitivity to NSNM. Furthermore, NSNM treatment causes rapid depletion of total glutathione and reduced glutathione. We also demonstrated that NSNM induces degradation of Sml1, a ribonucleotide reductase inhibitor involved in regulating dNTPs production. In summary, we define the various biological pathways targeted by isocyanates.

## Introduction

In recent decades, a key center of attention has been on understanding exposure to hazardous agents in the natural environment including water, soil and air. Elucidation of modes of action through studying effects of occupational exposure to environmental contaminants on human health is of foremost concern. Such exposures are often associated with a number of diseases, including cancer, diabetes, and infertility, etc. [1]. Evaluation of exposure to natural, environmental hazards, however, has not been properly elucidated in most human health risk assessments. Although preliminary evidence available in literature indicates that isocyanates and their derivatives may have deleterious health effects [2]–[4], the molecular mechanisms responsible for such an effect has never been addressed.

Isocyanates [5], a group of low molecular weight aromatic and aliphatic compounds containing a functional isocyanate group (-NCO), are essential raw materials with varied industrial applications [6]. Isocyanates are shown to be highly reactive to biological macromolecules leading to the formation of carbamylated products. These products include DNA cross links/adducts, which in turn contribute to cytotoxicity and harmful effects [7], [8]. Isocyanates are of interest in the field of genetic toxicology because they may act as electrophilic agents and react with DNA to produce genetic damage. It is also demonstrated that isocyanates are capable of undergoing bio-transformation reactions, such as DNA damage, apoptosis, oxidative stress, and inflammation in cultured human lymphocytes and lung fibroblasts [9], [10].

N-succinimidyl N-methylcarbamate (NSNM) is one of the carbamate esters which possess functional isocyanate group [11]. N-methyl carbamates are a group of organic compounds which share a common functional group with the general structure –NH(CO)O–. Also N-methylcarbamate esters are converted into isocyanates when the alcohol (–OH) group gets eliminated. To understand the mode of action of isocyanate we used NSNM and analysed its effect on budding yeast *Saccharomyces cerevisiae*. The budding yeast is considered as an attractive candidate for our studies because of its pliability to genetic manipulation and completely known genomic sequence [12]. Elucidation of the underlying mechanisms by which isocyanate shows its toxic effect will greatly facilitate the designing of improved precautionary measurement against these agents.

To understand the mode of action of isocyanate on yeast cells we screened the sensitivity of yeast mutants for genes involved in key cellular pathways including epigenetics, DNA damage checkpoint kinase, DNA repair and TOR pathways. Our results demonstrated that NSNM acts as an epigenetic modifier. Mitochondrial membrane potential and ROS levels were increased considerably following exposure to NSNM. Rapid depletion of intracellular glutathione was also observed upon treatment with NSNM. Taken together, our result shows that isocyanates target multiple biological pathways which contribute to its toxicity.

## Materials and Methods

### Strains, chemicals and growth media

The *Saccharomyces cerevisiae* strains used in this study are listed in Table S1. All chemicals used in this study, unless otherwise stated, were purchased from Sigma. N-succinimidyl N-methylcarbamate (Sigma Aldrich) was dissolved in DMSO to make 1 M stock solution. For making synthetic complete (SC) media all amino acids, YNB (yeast nitrogen base) and ammonium sulfate were mixed together following standard protocol [13]. All yeast strains were grown in SC media at 30°C. For solid Synthetic Complete Agar (SCA) media, 2% Bacto-agar was used in addition to components of SC liquid media.

### Growth assay and clonogenic cell survival assay

To investigate the biological effect of NSNM on the growth of yeast cells, growth assay was carried out by spot testing (as described earlier [14], [15]) using serial dilutions of mid-log phase cultures of wild-type and different mutant strains listed in Table S1. 3 μl of each undiluted and 10-fold serially diluted culture were spotted onto solid SCA plates containing vehicle DMSO or different concentrations of NSNM (100 and 200 μM). All plates were incubated at 30°C and growth of the yeast strains were recorded at periodic time intervals of 24, 48 and 72 h by scanning the plates using a HP scanner. For growth curve assays, exponentially growing yeast cells were treated with NSNM (5, 10, 20, 30, 50 and 100 μM) and optical density (OD_600_) was measured at regular intervals for 8 h. Clonogenic assay was carried as described earlier [16]. Equal number of mid-log phase cells from untreated (DMSO) or NSNM treated cultures (3 h) were spread on SCA plates. The plates were incubated at 30°C and survival was analyzed after 36 h of incubation by counting number of colonies per plate. Experiments were performed in triplicates and cell survival was calculated with respect to untreated cells.

### Micrococcal nuclease (MNase) assay

MNase assay was performed as described previously [17] with minor modifications. Exponentially growing yeast cells were treated with DMSO (control) or 100 μM NSNM for 3 hr. Equal number of cells were processed for MNase digestion. Spheroplasts were made from 100 ml cells and processed for MNase digestion as dilution series by diluting MNase in Digestion buffer (50 mM Tris pH 7.9, 5 mM CaCl_2_) to make 0, 50, 100, 200, 300, 500 and 1000 U/ml concentrations. MNase digestion was performed for 5 min at 37 °C and immediately reaction was stopped by adding stop solution (.25 M EDTA, 5% SDS). Phenol chloroform extraction was carried out, pellets were washed with 70% ethanol, air-dried, and re-suspended in 30 μl TE containing 100 μg/ml RNase. Samples were loaded on a 1.2% agarose gel and visualized by EtBr (Ethidium bromide) staining.

### Biochemical assay to investigate binding of NSNM with core histones

Core histones were purified from white leghorn chicken brain tissue as described earlier [18]. Free core histones (5 μg) were incubated separately with increasing concentrations of NSNM (0, 10, 20 and 40 μg) in 20 μl reaction volume (sodium phosphate buffer pH 7.2) at 37°C for 1 h as described earlier [13]. After incubation reaction mix were electrophoresed on 12% SDS-PAGE followed by immunoblotting with anti-H3 (Abcam, 1791) and anti-H4 antibody (Abcam, 15823).

### Detection of cellular ROS levels and assay for measuring mitochondrial membrane potential

To measure ROS production, we used 2, 7-dichlorodihydrofluorescein diacetate (DCF-DA) (Sigma, D6883). ROS and mitochondrial membrane potential were measured following protocol described previously [19]. Briefly, yeast cells were treated with 10 μM DCF-DA in culture media for 1 h prior to harvesting. Cells were washed twice in ice-cold PBS (phosphate buffer saline), resuspended in same buffer and immediately observed under fluorescence microscope (AXIOVERT 4.0) using FITC filter. The membrane potential-dependent stain MitoTracker (Molecular Probes- Invitrogen) was used to assess the mitochondrial membrane potential of yeast cells. After treatment with NSNM approximately 1×10^7^ yeast cells were harvested and washed with ice-cold PBS. Cells were resuspended in 100 μl of PBS followed by staining with MitoTracker. After staining, cells were visualized under fluorescence microscope (AXIOVERT 4.0) using Rhodamine filter.

### Glutathione measurement assays

Glutathione levels were measured using the method described earlier [19], [20]. Briefly, cells were grown to exponential phase and treated with DMSO (control) or NSNM (50 or 100 μM) for 3 h, washed with ice cold water, and resuspended in 250 μl of cold 1% 5-sulfosalicylic acid. Cells were broken by rigorous vortexing with glass beads and incubated at 4 °C for 15 min. The extracts were centrifuged and supernatants were used to determine glutathione levels. Total glutathione was determined by adding 10 μl of lysate to 150 μl of assay mixture (0.1 M potassium phosphate, pH 7.0, 1 mM EDTA, 0.03 mg/ml 5, 5′-dithiobis (2-nitrobenzoic acid), 0.12 unit of glutathione reductase). The samples were mixed and incubated for 5 min at room temperature followed by addition of 50 μl of NADPH (0.16 mg/ml). The formation of thiobis (2-nitrobenzoic acid) was measured spectrophotometrically at 420 nm over a 5-min period. Standard curves were generated for each experiment using 0–0.5 nmol of glutathione in 1% 5-sulfosalicylic acid. To measure GSSG alone, 100 μl lysate samples were derivatized by adding 2 μl of 97% 2-vinylpyridine, and the pH was adjusted by adding 2 μl of 25% triethanolamine followed by incubation at room temperature for 60 min. The samples were then assayed as described above for total GSSG. GSSG standards (0–0.1 nmol) were also treated with 2-vinylpyridine in an identical manner to the samples. Subtraction of the amount of GSSG in the lysate from the total glutathione concentration allowed a determination of GSH levels present in each sample.

### Flow cytometry analysis of yeast cells

Yeast cells in exponential phase were treated with alpha factor to synchronize cells in G1 phase as described earlier [19]. Cells were released in DMSO (control) or 50 μM NSNM containing media for 6 hours. Samples were collected at regular intervals and harvested by centrifugation and washed once with 50 mM sodium citrate buffer (pH 7.0). RNase A was added to the samples and incubated at 50°C for 1 h. RNase A-treated samples were transferred to BD FACS flow containing 20 mg/ml propidium iodide. Cellular DNA was detected by a BD FACS Aria III with BD FACS Diva software.

For ROS and mitochondrial membrane potential measurement, wild type yeast strain was grown till exponential phase in presence or absence of antioxidant agent GSH (10 mM) followed by treatment with indicated concentration of NSNM for three hours. Cells were stained with either MitoTracker or DCF-DA as described above before being analyzed by FACS.

### Confocal microscopy

To determine the effect of NSNM on Sml1 and Rad52, we analyzed Sml1-YFP or Rad52-YFP yeast strains by confocal microscopy as described previously [13]. A yeast colony was inoculated in 5 ml YPD and grown overnight at 30 °C. 200 μl of inoculate was transferred to fresh media and cells were grown till exponential phase. NSNM (50 μM) was added into culture media for 3 h. The cells were then harvested, washed twice with PBS, and finally dissolved in 200 μl PBS. The nuclear DNA was visualized with DAPI staining to a final concentration of 0.5 μg/ml. Confocal images were taken with LSM 780 confocal microscope (Carl zeiss, Germany) using appropriate filter combinations for either YFP fluorescence or DAPI. 63X oil immersion objective (Carl zeiss, Germany) was used for visualizing yeast cells. Images were processed via Zen 2010 software.

### Western blot analysis

Exponentially growing yeast cultures were treated with DMSO or NSNM for 3 h and harvested. Whole cell extracts were obtained from the yeast cell pellets by 20% trichloro acetic acid (TCA) precipitation following standard protocol [16]. The protein extracts were mixed with equal volume of 2X SDS-PAGE sample loading dye, boiled for 5 min, debris pelleted and the supernatant resolved by electrophoresis on a SDS-polyacrylamide gel. Immunoblotting was performed as described previously [16], [21]. IRDye® 800CW anti-Rabbit IgG (dilution 1∶15,000; LI-COR® Biosciences) was used as secondary antibody. Western blots were scanned by using Odyssey Infrared imager (LI-COR® Biosciences). Following primary antibodies were used: General H3 (Sigma, H0164), H3K4me1 (Abcam, 8895), H3K4me2 (Abcam, 32356), H3K9ac (Abcam, 69830), H3K9acS10P (Sigma, H9161), H3K18ac (Cell signaling, 9675), H3K27ac (Abcam, 45173), H3K36me3 (Sigma, SAB4800028), H4K8ac (Abcam, 15823), Rnr1 (Agrisera, ASO9 576), Rnr2 (Agrisera, ASO9 575), Sml1 (Agrisera, AS10 847), Polyclonal antibodies against recombinant TBP and RAP1 were raised in rabbit.

## Results

### NSNM is toxic to yeast cells in dose dependent manner

**Figure 1:**
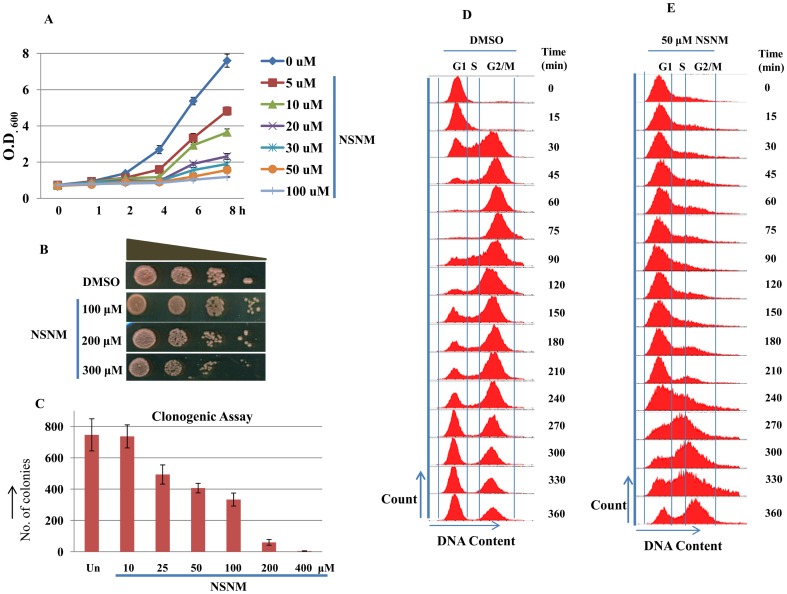
NSNM inhibits growth of wild type yeast cells in a dose-dependent manner. A) Growth curves of wild-type *S. cerevisiae* in the presence or absence of drug. Exponentially growing yeast cultures of the wild-type strain were treated with the indicated concentrations of NSNM. Growth was monitored by measuring OD_600_ at regular intervals for 8 h. B) Growth Assay; 1588-4C (Wild-type) was grown up to log phase. 3 μl of each undiluted and 10-fold serially diluted culture was spotted onto control SCA plates and SCA plates containing 100, 200 or 300 μM NSNM. All plates were incubated at 30°C for 72 h and photographed. C) Clonogenic assay; equal number of cells from mid-log phase of untreated and NSNM treated cultures (3 h) were spread on standard SCA plates in triplicate. All plates were incubated at 30°C and the colony forming ability was analyzed after 36 h. Number of colonies were counted and shown in the form of bar diagram. D and E) FACS analysis, showing the effect of the NSNM on yeast cell cycle. Wild-type cells were cultured in SC medium to exponential phase and treated with alpha factor to synchronize all cells in G1 phase. After synchronization cells were released in either DMSO (control) or 50 μM NSNM containing media. The culture was sampled at indicated time points and cellular DNA content was analysed by FACS.

In a pilot experiment, we tested the effect of NSNM on the growth of yeast cells. In liquid medium, significant growth inhibition was observed at very low dose of 5.0 μM NSNM and the growth was completely inhibited at 100 μM concentration (Fig. 1A). On solid SC medium, no significant growth inhibition was noticeable at 100 μM dose of NSNM (Fig. 1B). Hence, for experiments in solid media we have used relatively higher concentrations of NSNM (100, 200 and 300 μM) while in liquid cultures we used a maximum concentration of 100 μM. The growth-inhibitory activity of NSNM was further assessed by clonogenic assay. Wild type yeast cells were treated with different concentrations (0–100 μM) of NSNM for 3 h, followed by spreading equal number of cells on SCA plates. Plates were incubated for 36 h at 30°C and colonies were counted. These results showed that upon NSNM treatment yeast cells exhibited significant loss of viability in a dose-dependent manner (Fig. 1C).

Based on our growth curve experiments, we observed significant reduction in the growth rate of yeast cells upon NSNM treatment, suggesting that the NSNM is possibly causing defects in cell cycle. For confirmation, we performed FACS analysis to monitor cell cycle progression in the presence or absence of this chemical. Exponentially growing yeast cells were synchronized by alpha factor treatment for 2 h. After synchronization G1 arrested cells were released in 50 μM NSNM or DMSO (control) containing media. The DMSO treated cells moved to G2 phase within 60 min of release from alpha factor arrest (Fig. 1D) while NSNM inhibited the movement of cells to G2 phase for prolonged time (Fig. 1E). Even after 360 min of release in 50 μM NSNM containing media all cells were not able to progress to G2 phase suggesting that NSNM treatment causes delay in cell cycle progression.

### Screening of deletion mutants of key cellular pathways for NSNM sensitivity

**Figure 2:**
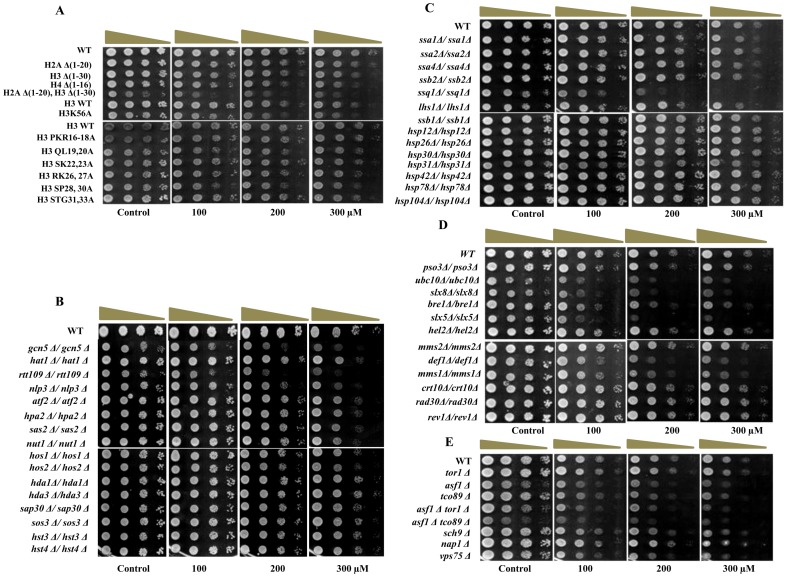
Screening of yeast deletion-mutants for NSNM sensitivity. A–E) Growth Assay; yeast deletion mutants of various pathways were grown up to log phase. 3 μl of each undiluted and 10-fold serially diluted culture was spotted onto control SCA plates and SCA plates containing 100, 200 and 300 μM NSNM. All plates were incubated at 30°C for 72 h and photographed. Mutant yeast strains of A) Histone tails, B) HATs and HDACs, C) Molecular chaperones, D) Protein-ubiquitination pathways, E) TOR pathway.

To elucidate the biological pathways targeted by NSNM we employed yeast deletion mutants. We screened the sensitivity of deletion-mutants of some of the key pathways of the cell including epigenetics, protein folding, TOR pathway, DNA damage checkpoint kinase, DNA repair and protein-ubiquitylation towards NSNM. Histone protein plays pivotal role in regulation of epigenetic information in the form of post-translational modifications. Most of the post-translational modifications occur in N-terminal tails of histones. Hence, we used genetic mutants of histone tails for checking their sensitivity to NSNM. To our surprise we did not observe any significant difference in growth of these mutants (Fig. 2A). Next, we investigated the effect of NSNM on mutant strains of histone modifying enzymes such as histone acetyl transferases (*gcn5Δ, hat1Δ, rtt109Δ, nlp3Δ, atf2Δ, hpa2Δ, sas2Δ* and *nut1Δ*) and histone deacetylases (*hos1Δ, hos2Δ, hst3Δ, hst4Δ, hda1Δ, hda3Δ, sap30Δ* and *sos3Δ*). Deletion of *GCN5* and *RTT109* exhibited enhanced growth inhibition (Fig. 2B) in response to NSNM treatment suggesting that it might affect histone acetylation status of the cells.

Next, we examined the effect of NSNM on molecular chaperones and protein-ubiquitylation pathways. We found that yeast genetic mutants of chaperone protein coding genes such as *ssa2Δ, ssa4Δ, ssb2Δ, hsp12Δ, hsp26Δ, hsp30Δ, hsp42Δ, hsp78Δ* and *hsp104Δ* did not display any sensitivity to NSNM (Fig. 2C). However, the deletion mutants of ubiquitylation factors (*ubc10Δ, slx8Δ, hex3Δ, mms1Δ, def1Δ*) were found to be hypersensitive for NSNM (Fig. 2D). Interestingly, while screening several other mutants, we encountered Ssq1 (mitochondrial hsp70-type chaperone) [22] deletion mutant to be hypersensitive (Fig. 2C) which suggests that it specifically targets a mitochondrial chaperone. Furthermore, we also investigated the effect of NSNM on TOR pathway. The highly conserved protein kinase TOR and its signaling network controls cell growth in response to nutrients, growth factors, and other environmental conditions [23]. We observed significant sensitivity of some of the mutants of TOR pathway such as *asf1Δ, tco89Δ*, and *vps75Δ* (Fig. 2E). The growth inhibitory effect of NSNM was further increased when two mutations were combined as in *asf1Δ tor1Δ* and *asf1Δ tco89Δ*. Altogether, through genetic screening of yeast mutants of various pathways, we for the first time in yeast found that NSNM targets multiple biological pathways.

### NSNM treatment causes alteration in histone modifications and formation of histone adducts

**Figure 3:**
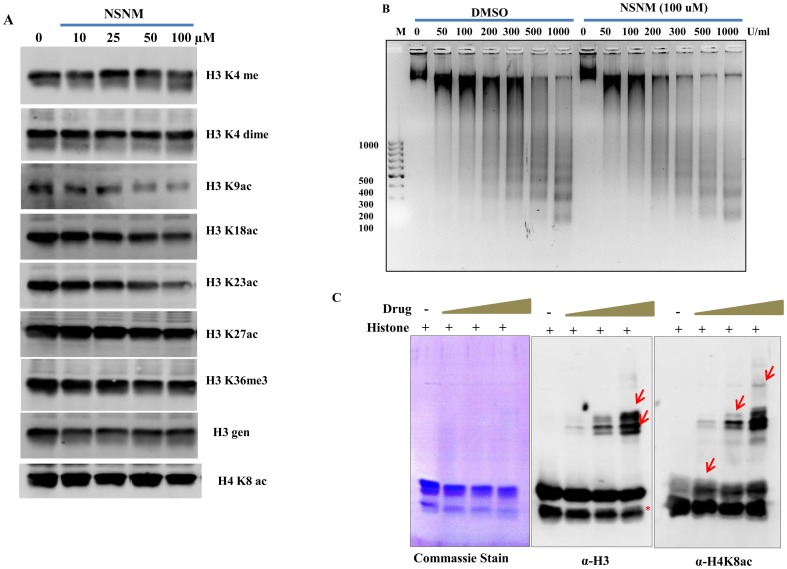
NSNM induces alterations in histone modifications and makes adduct with histone H3 and H4. A) Wild-type (1588-4C) cells were cultured up to log phase and treated with increasing concentrations of NSNM (0, 10, 25, 50, 100 μM) for 3 hr. Whole cell extracts were prepared by TCA extraction method and samples were subjected to western blot analysis using indicated antibodies. B) Wild type yeast cells were treated with DMSO and 100 μM NSNM for 3 h at OD_600_ (0.8) and equal numbers of cells were processed for MNase digestion. MNase was used at different concentrations (0, 50, 100, 200, 300, 500 and 1000 U/ml). Samples were run on 1.2% agarose gel, together with DNA ladder, and stained with ethidium-bromide. C) Core histones were incubated with increasing concentrations of NSNM for 1 h at 37 °C. Proteins were resolved by 12% SDS-PAGE. The interaction of NSNM with histones was analyzed by probing with histone H3 and H4 antibodies. Arrow indicates the appearance of high molecular weight band detected by H3 and H4 antibody respectively. * represents non-specific band detected by H3 antibody.

Our genetic screening results motivated us to examine the effect of NSNM on epigenetics. Histones play pivotal role in carrying epigenetic information in the form of post-translational modifications. To observe its effect on epigenetics, we analyzed global histone modifications with NSNM treatment in wild type yeast cells. We found a decrease in most of the histone acetylation marks in a dose-dependent manner (Fig. 3A). H3K9ac, H3K18ac, H3K23ac, H3K27ac were decreased while there was no change in methylation marks upon NSNM treatment (Fig. 3A). Modulation of the acetylation status of histones is an important mechanism for regulating gene expression and chromatin structure. Since, we observed a decrease in histone acetylation upon NSNM exposure, we analyzed its effect on chromatin architecture. We performed MNase assay that provides information regarding global chromatin structure. However, we did not detect any obvious alteration in chromatin structure (Fig. 3B) suggesting that NSNM does not affect global chromatin architecture; it targets specific regions of the genome. All together, these results suggest that NSNM acts as an epigenetic modifier; further studies should be performed to elucidate its effect on host epigenetics.

Isocyanates have the potential to spontaneously bind with biological macromolecules leading to the formation of products such as DNA cross links/adducts, which in turn contribute to cytotoxicity and harmful effects [7], [8]. These observations led us to propose that this chemical might also react with histones. To investigate whether NSNM binds to histones, we incubated pure histones with NSNM compound (Fig. 3C). We observed an adduct formation with histone H3 and H4 as detected by immunoblotting using H3 and H4 antibodies respectively.

### NSNM causes rapid depletion of intracellular glutathione, increase in the mitochondrial membrane potential and intracellular ROS levels

**Figure 4:**
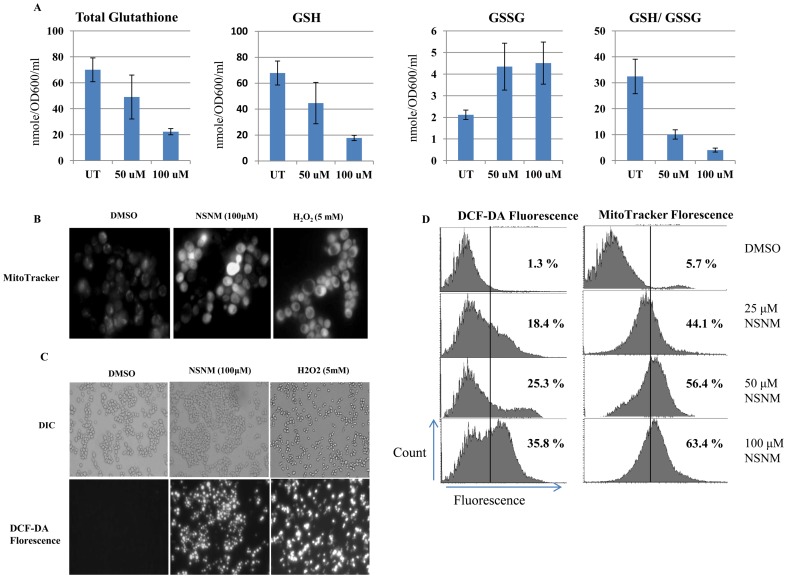
NSNM causes depletion in total glutathione levels and increase intracellular ROS levels. (A) Wild type cells were grown in DMSO or indicated concentration of NSNM for 3 h. GSH, GSSG, and the GSH: GSSG ratios were determined. Values are means S.D. of three independent cultures. (B & C) Mitochondrial membrane permebility and reactive oxygen species (ROS) production detected by MitoTracker and DCF-DA respectively in control cells and cells treated with 50 or 100 μM of NSNM for 3 h. Cells treated with 5 mM H_2_O_2_ served as positive control. Upper panel of (C) shows phase contrast microscopic images; the lower panel show florescence microscopic image of the same cells. (D) Wild-type yeast strain grown in SC media and treated with increasing concentration of NSNM (25, 50 or 100 μM) for 1 3 h. Yeast cells were processed for FACS analysis after staining with either DCF-DA or MitoTracker Red. % value depicts the proportion of cells showing fluorescence after staining with indicated dyes.

To understand the molecular mechanism behind growth inhibition caused by NSNM we measured total glutathione, GSH and GSSG levels upon NSNM treatment. Oxidative stress is indicated by alterations in total glutathione, GSH and GSSG levels, and a decrease in GSH: GSSG ratio [20]. We calculated these parameters and compared wild type cells grown in DMSO (control) with cells grown in 50 μM or 100 μM NSNM. Comparison of untreated versus the NSNM treated samples indicated that the treatment causes alterations in oxidative stress parameters. Upon NSNM treatment total glutathione and reduced glutathione (GSH) were decreased drastically while oxidized glutathione (GSSG) was elevated relative to untreated cells (Fig. 4A), consequently the ratio of the GSH: GSSG level was reduced after NSNM treatment (Fig. 4A).

The alterations in oxidative parameters indicate that NSNM might be affecting the cellular redox status. We used MitoTracker orange to examine the mitochondrial membrane potential. As shown in Fig.4B, MitoTracker staining could be imaged without NSNM treatment, but it showed very low intensity. However, with NSNM exposure, intense MitoTracker staining was observed. A similar increase in mitochondrial membrane potential was observed with 5.0 mM of H_2_O_2_ (Fig. 4B). These results suggest that NSNM targets energy metabolism process of host cells by interfering with mitochondrial functioning. Increase in mitochondrial membrane potential is often associated with an increase in cellular ROS levels. Next, we tried to delineate the intracellular molecular targets of NSNM, aiming to understand the molecular insights of ROS generation upon treatment with this compound. Exponentially growing yeast cells were treated with 100 μM NSNM for 3 h. Hydrogen peroxide treatment served as a positive control for ROS increase. Remarkably, NSNM induced an increase in ROS generation, which was revealed by DCF-DA staining (Fig. 4C). These results indicate that NSNM potentially inhibits the growth of wild type *S. cerevisiae* in ROS-dependent manner. To further confirm these results, we performed FACS analysis of yeast cells after treating them with 25, 50 or 100 μM NSNM for 3 h. Cells were either stained with DCF-DA or MitoTracker and analyzed through FACS to quantify the fluorescence of these dyes. Both DCF-DA and MitoTracker fluorescence was increased in a dose-dependent manner (Fig. 4D). Altogether these results suggested that NSNM causes elevation of oxidative stress in budding yeast.

### *sod1*/*sod2*-deletion causes increased sensitivity to NSNM while supplementation of reduced glutathione (GSH) rescues the effect

**Figure 5:**
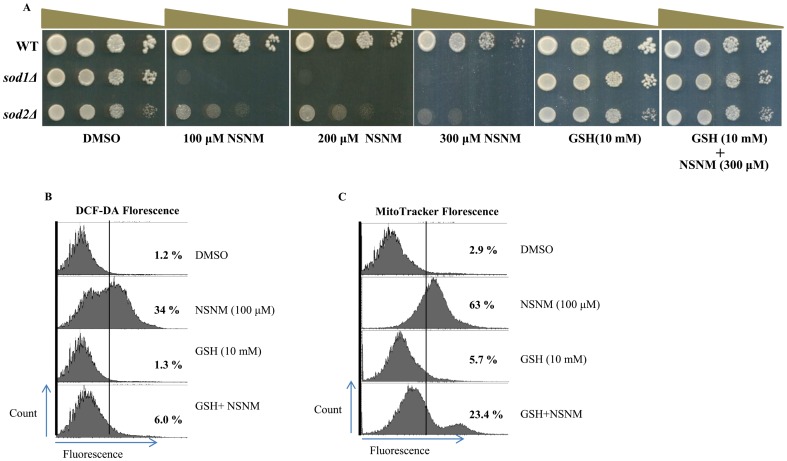
SODs deletions are hypersensitive to the NSNM while GSH supplementation rescues the effect. (A) Growth Assay; Wild type, *sod1Δ* or *sod2Δ* was grown up to log phase. 3 μl of each undiluted and 10-fold serially diluted culture was spotted onto control SCA plates, SCA plates containing 100, 200, 300 μM NSNM, and SCA plates impregnated with GSH (10 mM) in combination with 300 μM NSNM. All plates were incubated at 30°C for 72 h and photographed. (B &C) Wild-type yeast strain grown in SC media supplemented with or without 10 mM GSH for 1 h followed by exposure to 100 μM NSNM for 3 h. Yeast cells were processed for FACS analysis after staining with either DCF-DA (B) or MitoTracker Red (C). % value depicts the proportion of cells showing florescence after staining with indicated dyes.

Superoxide dismutases (SODs) are a class of enzymes that catalyze the dismutation of superoxide into hydrogen peroxide and oxygen. In budding yeast SODs have two forms *SOD1* and *SOD2*. It is one of the main anti-oxidative enzymes against reactive oxygen species (ROS), which are generated during different stress conditions. To investigate the possible role of Sod1 or Sod2 for conferring resistance to NSNM, we compared the growth of *sod1Δ/sod2Δ* and wild type cells in the presence of increasing dose of NSNM. As shown in Fig. 5A, *sod1Δ/sod2Δ* mutants were hypersensitive to NSNM. Because Sod1 or Sod2 are primarily antioxidant enzymes, we sought to investigate whether the increased sensitivity of *sod1Δ/sod2Δ* for NSNM is due to increased ROS levels. We analyzed the effect of the antioxidants, such as reduced glutathione (GSH), on the growth phenotype of *sod1Δ/sod2Δ*. As shown in Fig. 5A, the addition of the 10 mM GSH restored the growth of *sod1Δ/sod2Δ* in the presence of NSNM suggesting the role of ROS behind the toxicity exhibited by NSNM. Furthermore, to determine whether the ROS accumulation in NSNM treated cells is facilitated by increase in free radicals, we investigated the effect of NSNM on ROS levels in presence of GSH by FACS. Interestingly, the increase in ROS levels (Fig. 5B) and mitochondrial membrane potential (Fig. 5C) due to NSNM treatment recovered back to the normal levels when the medium was supplemented with 10 mM GSH.

Taken together, these results indicate that NSNM causes generation of reactive oxygen species leading to growth inhibition/death of yeast cells.

### NSNM causes degradation of Sml1 without RNR induction or Rad52 foci formation

**Figure 6:**
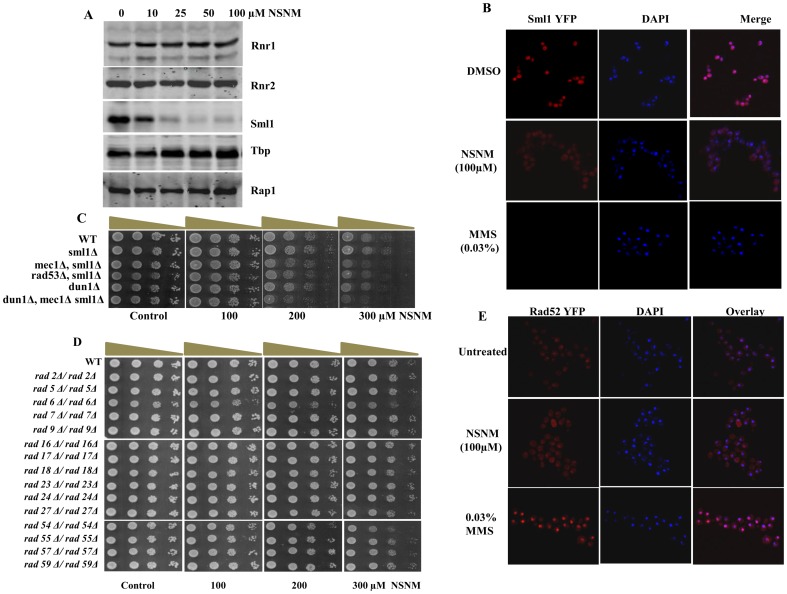
NSNM exposure leads to degradation of Sml1 without activating RNR genes or Rad52 foci formation. (A) Whole cell extracts were prepared by TCA extraction method and samples were subjected to western blot analysis with indicated antibodies. Blotting with antibodies against Tbp and Rap1 or Ponceau S staining of representative blot were used as loading controls. (B) Sml1-YFP tagged strain were treated with NSNM (100 μM) for 3 h. For control same strains were treated with MMS (0.03%), images were taken as described in materials and methods. (C and D) Growth Assay; wild type and different mutant yeast strains were spotted onto control SCA (DMSO) plates or SCA plates containing 100, 200 or 300 μM NSNM. All plates were incubated at 30°C for 72 h and photographed. (E) NSNM exposure does not lead to Rad52 foci formation. Rad52-YFP tagged yeast strain was treated with 100 μM NSNM for 3 h, same strain was treated with 0.03% MMS (control) prior to visualization of foci by confocal microscopy.

Our results demonstrated that NSNM significantly increases intracellular ROS levels and mitochondrial membrane potential that are hallmarks of oxidative stress [24]. Next, we proposed that NSNM induces ROS generation that might lead to DNA damage. The Mec1-Rad53-Dun1 DNA damage and replication checkpoint kinase cascade is responsible for promoting yeast RNR activity in response to DNA damage or DNA replication stress. Mec1-Rad53-dun1 kinases are activated upon DNA damage leading to activation of RNR genes and degradation of Sml1 [25]–[27]. Our western blot analysis of Rnr1 and Rnr2 revealed that treatment with NSNM did not induce their expression (Fig. 6A). Surprisingly, significant degradation of Sml1 was observed in yeast cells following NSNM treatment (Fig. 6A). To further confirm Sml1 degradation, we performed confocal microscopy. We used Sml1-YFP tagged cells pre-treated with NSNM for 3 hr and did not observe any fluorescence as opposed to untreated cells (Fig. 6B). 0.03% Methyl methanesulfonate (MMS) treated cells were used as a positive control. Next, we analyzed the effect of NSNM on DNA damage checkpoint mutants. We failed to observed growth inhibition of *mec1Δ, rad53Δ*, and *dun1Δ* deletion mutants in presence of NSNM (Fig. 6C) suggesting that this compound is not targeting DNA damage checkpoint pathway directly.

To gain additional insight into the mechanisms by which DNA repair and damage tolerance pathways may be activated upon NSNM induced DNA damage, we examined the sensitivity of strains defective in different DNA damage repair pathways. To understand the specific effects of NSNM, we employed various yeast knockout mutant strains defective in DSBR (double-strand break repair), NER (Nucleotide Excision Repair) and BER (Base Excision Repair). Mutants defective in DNA single or double-strand break repair pathways (*rad5Δ, rad6Δ, rad9Δ, rad17Δ, rad18Δ, rad24Δ, rad27Δ, rad54Δ, rad55Δ, rad57Δ, rad59Δ*), NER and BER (*rad2Δ, rad7Δ, rad16Δ, rad23Δ*) pathways did not show any significant sensitivity to NSNM (Fig. 6D). Rad52 foci were not observed upon NSNM treatment (Fig. 6E) suggesting that it does not target DNA damage repair pathways. The key role played by Rad52 has been described by its ability to mediate annealing of homologous DNA strands [28]–[32]. We used the Rad52-YFP strain to visualize foci formation upon NSNM treatment, and as a positive control we treated cells with MMS (0.03%). Altogether, our results demonstrated that NSNM causes partial activation of DNA damage checkpoint kinase pathway (Sml1 degradation) but it does not induce expression of RNR genes and Rad52 foci formation.

## Discussion

In the present study we used budding yeast *Saccharomyces cerevisiae* to understand the mechanism behind the toxicity exhibited by the isocyanates. In this work, we utilized a non-toxic surrogate chemical equivalent of isocyanate (NSNM) to study the response in yeast cells. We screened some of important pathways including epigenetics, histone modifications, DNA damage response, protein-ubiquitylation and chaperones, oxidative stress, TOR pathway and DNA repair pathways. Through screening we identified few molecules including Gcn5, Rtt109, Ssq1 and Slx5 which are required to provide tolerance to NSNM.

One of the important findings of the current study is that NSNM acts as an epigenetic modifier. It makes adduct with histone and its treatment causes decrease in the global histone acetylation. Change in epigenetics or alteration in histone modifications occurs during regulation of various biological processes [33], [34]. One of the mechanism by which some of the small molecules/drug show their effect is through directly reacting with chromatin. Similar histone adduct formations have been observed with several compounds such as acrolein [35], RAPTA-C [36], KP1019 [13] etc. Now it is clear from various studies that small molecules can alter epigenetic profile of an organisms, such as DNA methylation, chromatin modifications, and non-coding RNAs leading to the alteration in chromatin structure and gene expression [37]. From our genetic screening we found that deletion mutants of two of the histone acetyltransferases *GCN5* and *RTT109* showed enhanced growth inhibition in response to NSNM treatment suggesting that it might target epigenetic machinery of the cells. Additionally, we observed a decrease in histone acetylation marks upon treatment with this compound, suggesting that the adduct formation might be the mechanism responsible for the decrease in histone acetylation. Based on our present study we can propose that NSNM reacts with histones to make adducts, therefore compromising downstream processes that may represent a vital mechanism by which NSNM influences its biological functions.

NSNM is a reactive compound and based on our sensitivity assays we observed that it is targeting multiple cellular pathways in yeast. Next, we measured the cellular redox status upon treatment with NSNM. Interestingly, we detected increase in mitochondrial membrane potential as revealed by MitoTracker staining. Increase in mitochondrial membrane potential is known to increase intracellular ROS levels [38], and consistent with this, we also observed enhanced ROS levels upon exposure to NSNM which are hallmarks of oxidative stress [24]. Oxidative stress has been proved to be a type of stimulus that can elicit stress-response signal-transduction pathways [39]. It is widely recognized that free radicals or ROS are involved in speeding up the aging process or shortening the life span [40]. At elevated concentrations, ROS exert deleterious consequences on various cellular pathways. Through these pathways cells can activate mitochondrial activity leading to the overproduction of toxic ROS. Cells are equipped with enzymatic and nonenzymatic antioxidant systems to eliminate ROS/RNS and maintain redox homeostasis [41]–[43]. A major class of enzymatic antioxidants, which catalyze the dismutation of O_2_^−^ to H_2_O_2_ are the superoxide dismutases (SODs) [44]. Further conversion of H_2_O_2_ to H_2_O+O_2_ occurs through the action of catalase, a heme-based enzyme that is normally localized in the peroxisome [45]. Deletion of SODs enhances the growth inhibitory effect of NSNM (Fig. 5A). H_2_O_2_ is converted to O_2_ through coupled reactions, with the conversion of reduced glutathione (GSH) to oxidized glutathione (GSSG), catalyzed by glutathione peroxidase (GPX) [46]–[48]. Our results demonstrated that NSNM treatment causes alteration in oxidative stress parameters, the oxidized glutathione (GSSG) was increased significantly while the total glutathione was reduced following NSNM exposure. These results suggest that NSNM treatment causes oxidative stress.

Oxidative stress is often associated with induction of DNA damage response (DDR) [49]. Furthermore, we tested the effect of NSNM on DDR by analyzing its effect on DNA damage checkpoint kinase pathway. Our results showed that NSNM treatment causes drastic decrease in Sml1 levels (Fig. 6A). Sml1 acts as an inhibitor of Rnr1, a member of RNR complex which synthesizes dNTPs [27]. Degradation of Sml1 facilitates the production of dNTPs by the RNR complex [25]. We propose that NSNM induced degradation of Sml1 will favor the synthesis of dNTPs. It has been previously described that the cells lacking Sml1 are reported to have higher levels of dNTPs, independent of the increase in RNR transcription [50], [51]. Similar results have also been reported upon treatment with curcumin [16]. There are some reports which explains that isocyanates can react with the exocyclic amino group of dNTPs including deoxycytidine, deoxyadenosine and deoxyguanosine to produce carbamoylated products [52], [53], suggesting that it might affect *in vivo* cellular dNTPs pool. Although, degradation of Sml1 upon exposure to NSNM partly supports our hypothesis, its effect on cellular dNTPs levels needs to be further tested.

Altogether, we have illustrated the key pathways affected by isocyanates. Our findings suggest that the toxic effects of isocyanate are largely contributed by cumulative effect of alteration in redox status and epigenetic modifications. Thus, it is likely that many lethal challenges by isocyanates are the result of hijacking natural stress-response machineries to induce ROS production above threshold levels leading to cell death. Our studies undoubtedly provide important insights into the physiological pathways affected by isocyanates. Further analysis on global and gene-specific changes in response to isocyanates will provide more insights into the mechanisms of toxicity exhibited by them. We anticipate that these facts would assist to devise improved approaches in assessment of toxicity caused by isocyanates.

## Supporting Information

Table S1 List of yeast strains used in this study.
(DOCX)
