# Correction: Assessment of the Biological Pathways Targeted by Isocyanate Using N-Succinimidyl N-Methylcarbamate in Budding Yeast *Saccharomyces cerevisiae*

**DOI:** 10.1371/journal.pone.0306937

**Published:** 2024-07-05

**Authors:** Gajendra Kumar Azad, Vikash Singh, Raghuvir S. Tomar

After publication of this article [1], concerns were raised about Figs 2 and 6, which the authors address with this Correction.

**Fig 2:**
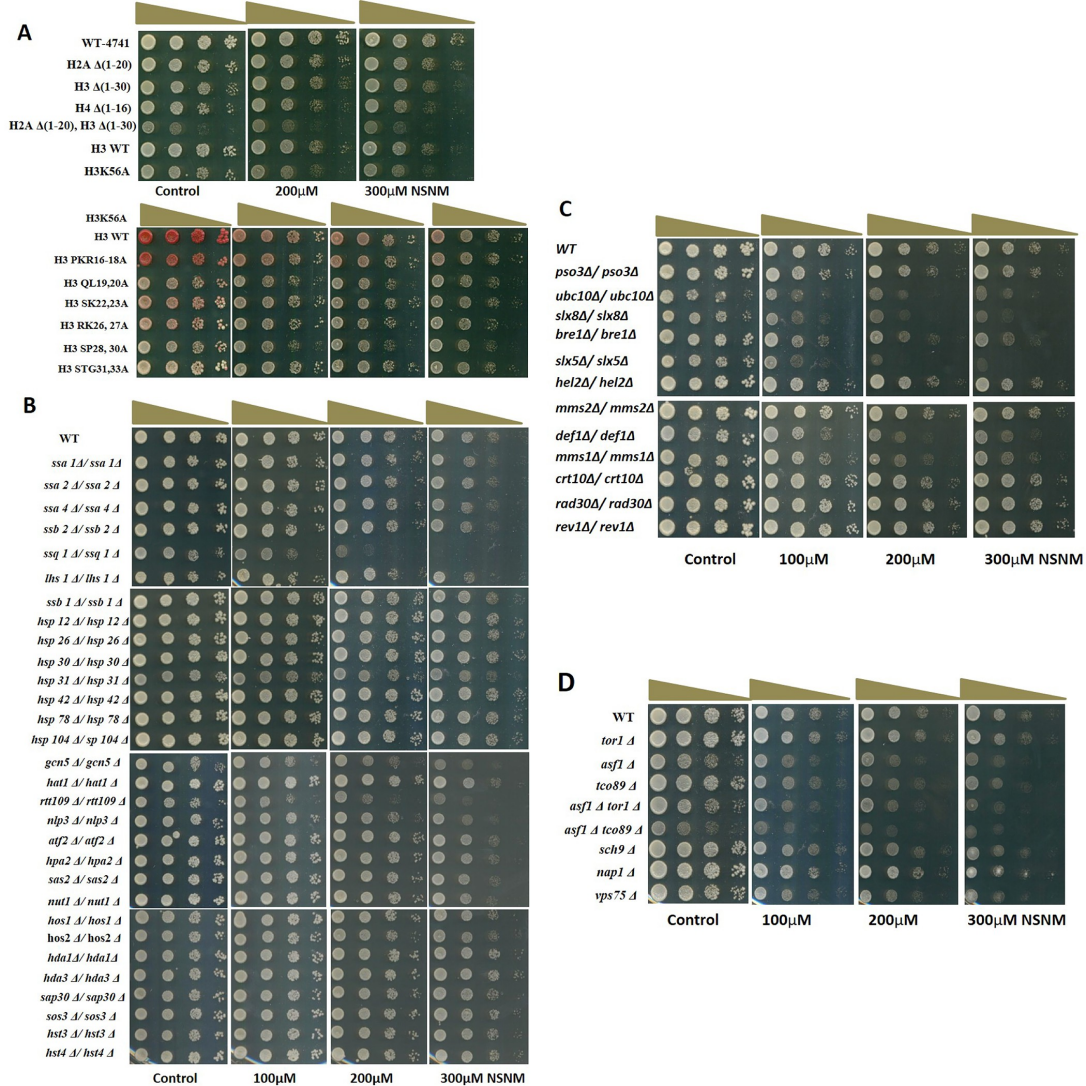
Screening of yeast deletion-mutants for NSNM sensitivity. A–D) Growth Assay; yeast deletion mutants of various pathways were grown up to log phase. 3 μl of each undiluted and 10-fold serially diluted culture was spotted onto control SCA plates and SCA plates containing increasing concentration of NSNM (mentioned in each panel). All plates were incubated at 30 ⁰C for 72 h and photographed. Mutant yeast strains of A) Histone tails, B) HATs, HDACs, and Molecular chaperones, C) Protein-ubiquitination pathways, D) TOR pathway.

Specifically, errors were made in the preparation of Fig 2 as follows:

- In the top row of Fig 2A, the Control and 100µM panels are incorrect.
- In Fig 2D, both the upper and lower 300µM panels are incorrect and are duplicates of the 200µM panels.

The authors also clarify that the same WT Control colonies were used for Fig 2B and 2C, as a shared control.

Here the authors provide a revised Fig 2 in which the incorrect panels are replaced with the correct images from the original experiments. In the revised Fig 2, mutant yeast strains of HATs and HDACs and of molecular chaperones (shown separately in parts 2B and 2C respectively in the originally published figure) are presented together in part 2B with a single shared WT row. The panels showing mutant yeast strains of protein-ubiquitination pathways and TOR pathway are relabeled as Fig 2C and 2D, respectively.

To reflect the updated labelling of the Figure parts, the correct text for the second paragraph of the Results subsection titled Screening of deletion mutants of key cellular pathways for NSNM sensitivity is as follows:

Next, we examined the effect of NSNM on molecular chaperones and protein-ubiquitylation pathways. We found that yeast genetic mutants of chaperone protein coding genes such as *ssa2Δ*, *ssa4Δ*, *ssb2Δ*, *hsp12Δ*, *hsp26Δ*, *hsp30Δ*, *hsp42Δ*, *hsp78Δ* and *hsp104Δ* did not display any sensitivity to NSNM (Fig 2B). However, the deletion mutants of ubiquitylation factors (*ubc10Δ*, *slx8Δ*, *hex3Δ*, *mms1Δ*, *def1Δ*) were found to be hypersensitive for NSNM (Fig 2C). Interestingly, while screening several other mutants, we encountered Ssq1 (mitochondrial hsp70-type chaperone) [22] deletion mutant to be hypersensitive (Fig 2B) which suggests that it specifically targets a mitochondrial chaperone. Furthermore, we also investigated the effect of NSNM on TOR pathway. The highly conserved protein kinase TOR and its signaling network controls cell growth in response to nutrients, growth factors, and other environmental conditions [23]. We observed significant sensitivity of some of the mutants of TOR pathway such as *asf1Δ*, *tco89Δ*, and *vps75Δ* (Fig 2D). The growth inhibitory effect of NSNM was further increased when two mutations were combined as in *asf1Δ tor1Δ* and *asf1Δ tco89Δ*. Altogether, through genetic screening of yeast mutants of various pathways, we for the first time in yeast found that NSNM targets multiple biological pathways.

It is additionally noted that the MMS (0.03%) Sml1 YFP panel in Fig 6B of [1] appears devoid of background signal when color levels are adjusted. The corresponding author noted that Sml1 degradation in response to DNA damage is a well-known phenomenon and following consultation with a *PLOS ONE* Editorial Board Member, this issue is considered resolved.

The original uncropped images underlying the updated Fig 2 and Fig 6 in [1] are available in the S1 File provided with this notice. The original raw image for Fig 6A was unavailable. The corresponding author stated that the original, uncropped images underlying this article [1] are partly available for Figs 1, 3, 4 and 5, and that the individual-level quantitative data underlying this article [1] are available for Figs 1A, 1C and 4A.

The authors apologize for the errors in the published article.

## Supporting information

S1 File Uncropped images of Figs 2 and 6. This file includes the uncropped images for the updated Fig 2 and for Fig 6 in [1].
(PDF)
