# Supplementary material for: Assessment of the Biological Pathways Targeted by Isocyanate Using N-Succinimidyl N-Methylcarbamate in Budding Yeast Saccharomyces cerevisiae
Source: PLoS One. 2014 Mar 24;9(3):e92993. doi: 10.1371/journal.pone.0092993 (PMC3963962; doi:10.1371/journal.pone.0092993)
Supplement: Table S1 — List of yeast strains used in this study. (DOCX) [file pone.0092993.s001.docx]

**Table S1.**

| S.No. | Strain Name | Genotype | Mutation | Source/Lab |
| --- | --- | --- | --- | --- |
| 1 | WT-15884C | MATa ade2-1 can1-100 his3-11,15 leu2-3,112 trp1-1 ura3-1 | WT | Toshi Tsukiyama |
| 2 | H2A(1-20) | MATa his3-1 leu2-0 met15-0 ura3-0 hht1-hhf1::KAN hhf-2hht2::NAT hta1-htb1::HPH hta2-htb2::NATp[CEN LEU2 hta1Δ(1-20)-HTB1-HHT2-HHF2] | H2AΔ(1-20) | C.D. Allis |
| 3 | H3(1-30) | MATa his3-1 leu2-0 met15-0 ura3-0 hht1-hhf1::KAN hhf-2hht2::NAT hta1-htb1::HPH hta2-htb2::NATp[CEN LEU2 HTA1-HTB1-hht2Δ(1-30)-HHT2-HHF2] | H3Δ (1-30) | C.D. Allis |
| 4 | H4(1-16) | MATa his3-1 leu2-0 met15-0 ura3-0 hht1-hhf1::KAN hhf-2hht2::NAT hta1-htb1::HPH hta2-htb2::NATp[CEN LEU2 HTA1-HTB1-HHT2-hhf2 Δ(1-16)] | H4Δ (1-16) | C.D. Allis |
| 5 | H2A Δ(1-20), H3 Δ(1-30) | MATa his3-1 leu2-0 met15-0 ura3-0 hht1-hhf1::KAN hhf-2hht2::NAT hta1-htb1::HPH hta2-htb2::NATp[CEN LEU2 hta1Δ(1-20)-HTB1- hht2 Δ(1-30)-HHF2] | H2A Δ(1-20), H3 Δ(1-30) | C.D. Allis |
| 6 | H3 WT | MATa his3Δ200 leu2Δ0 lys2Δ0 trp1Δ63 ura3Δ0met15Δ0 can1:: MFA1pr-HIS3 hht1-hhf1:: NatMX4 hht2-hhf2:: [HHTS-HHFS]-URA3 | WT | R. Nicholas Laribee |
| 7 | H3K56A | *H3 WT; [H3K56A]-URA3* | H3K56A | R. Nicholas Laribee |
| 8 | WZY 42 WT | MATa, ura3-52, leu2D1, trp1D63, his3D200, lys2-801, ade2-101, hht1-hhf1::pWZ405-F2F9-LEU2,hht2- hf2::pWZ403-F4F10-HIS3, YCp50-copyII (HHT2-HHF2 | WT | Eun-Jung Cho |
| 9 | H3 PKR16-18A | WZY 42; pWZ414-F13:: H3 PKR16-18A | H3 PKR16-18A | Eun-Jung Cho |
| 10 | H3 QL19,20A | WZY 42; pWZ414-F13:: H3 QL19,20A | H3 QL19,20A | Eun-Jung Cho |
| 11 | H3 SK22,23A | WZY 42; pWZ414-F13:: H3 SK22,23A | H3 SK22,23A | Eun-Jung Cho |
| 12 | H3 RK26, 27A | WZY 42; pWZ414-F13:: RK26, 27A | H3 RK26, 27A | Eun-Jung Cho |
| 13 | H3 SP28, 30A | WZY 42; pWZ414-F13:: SP28, 30A | H3 SP28, 30A | Eun-Jung Cho |
| 14 | H3 STG31-33A | WZY 42; pWZ414-F13:: STG31-33A | H3 STG31-33A | Eun-Jung Cho |
| 15 | *sml1Δ* | MATa ade2-1 can1-100 his3-11,15bleu2-3, 112 trp1-1 ura3-1Sml1Δ::HIS3 | *sml1Δ* | Andrei Chabes |
| 16 | *mec1Δ, sml1Δ* | MATa ade2-1 can1-100 his3-11,15bleu2-3, 112 trp1-1 ura3-1 mec1Δ::TRP1 sml1Δ::HIS3 | *mec1Δ, sml1Δ* | Andrei Chabes |
| 17 | *rad53Δ, sml1Δ* | MATa ade2-1 can1-100 his3-11,15bleu2-3, 112 trp1-1 ura3-1 rad53Δ::HphMX4 sml1Δ::HIS3 | *rad53Δ, sml1Δ* | Andrei Chabes |
| 18 | *dun1Δ* | MATa ade2-1 can1-100 his3-11,15bleu2-3, 112 trp1-1 ura3-1 dun1Δ::KanMX6 | *dun1Δ* | Andrei Chabes |
| 19 | *mec1Δ, sml1Δ, dun1 Δ* | MATa ade2-1 can1-100 his3-11,15bleu2-3, 112 trp1-1 ura3-1 mec1Δ::TRP1 sml1Δ::HIS3 dun1 Δ::KanMX6 | *mec1Δ, sml1Δ, dun1 Δ* | Andrei Chabes |
| 20 | Sml1-YFP | Mat a ADE2 YFP-SML1 CFP-RNR1 RAD5 | YFP-SML1 | Rodney Rothstein |
| 21 | Rad52-YFP | Mat a ADE2 trp1-1 LYS2 RAD52-YFP RAD5;Isogenic to W303 | RAD52-YFP | Michael Lisby |
| 22 | WT 4743 | MATa/α his3Δ1/his3Δ1 leu2Δ0/leu2Δ0 LYS2/lys2Δ0 met15Δ0/MET15 ura3Δ0/ura3Δ0 | WT | Yeast deletion collection-Open Biosystems(YDC-OB) |
| 23 | *hos1Δ/hos1Δ* | Isogenic to BY4743  *hos2Δ::KANMX4* | *hos1Δ* | YDC-OB |
| 24 | *hos2Δ/hos2Δ* | Isogenic to BY4743  *hos2Δ::KANMX4* | *hos2Δ* | YDC-OB |
| 25 | *hda1Δ/hda1Δ* | Isogenic to BY4743  *hda1Δ*::KANMX4 | *hda1Δ* | YDC-OB |
| 26 | *hda3Δ/hda3Δ* | Isogenic to BY4743  *hda3Δ*::KANMX4 | *hda3Δ* | YDC-OB |
| 27 | *sap30Δ/ sap30Δ* | Isogenic to BY4743 *sap30Δ*::KANMX4 | *sap30Δ* | YDC-OB |
| 28 | *sos3Δ/ sos3Δ* | Isogenic to BY4743 *sos3Δ*::KANMX4 | *sos3Δ* | YDC-OB |
| 29 | *hst3Δ/ hst3Δ* | Isogenic to BY4743  *hst3Δ*::KANMX4 | *hst3Δ* | YDC-OB |
| 30 | *hst4Δ/ hst4Δ* | Isogenic to BY4743 *hst4Δ*::KANMX4 | *hst4Δ* | YDC-OB |
| 31 | *gcn5Δ/gcn5Δ* | Isogenic to BY4743  *gcn5Δ*::KANMX4 | *gcn5Δ* | YDC-OB |
| 32 | *hat1Δ/hat1Δ* | Isogenic to BY4743  *hat1Δ*::KANMX4 | *hat1Δ* | YDC-OB |
| 33 | *rtt109Δ/rtt109Δ* | Isogenic to BY4743  *rtt109Δ*::KANMX4 | *rtt109Δ* | YDC-OB |
| 34 | *nlp3Δ/nlp3Δ* | Isogenic to BY4743 *nlp3Δ*::KANMX4 | *nlp3Δ* | YDC-OB |
| 35 | *atf2Δ/atf2Δ* | Isogenic to BY4743 *atf2Δ*::KANMX4 | *atf2Δ* | YDC-OB |
| 36 | *hpa2Δ/ hpa2Δ* | Isogenic to BY4743  *hpa2Δ*::KANMX4 | *hpa2Δ* | YDC-OB |
| 37 | *sas2Δ/ sas2Δ* | Isogenic to BY4743  *sas2Δ*::KANMX4 | *sas2Δ* | YDC-OB |
| 38 | *nut1Δ/nut1Δ* | Isogenic to BY4743 *nut1Δ*::KANMX4 | *nut1Δ* | YDC-OB |
| 39 | *rad2Δ/ rad2Δ* | Isogenic to BY4743 *rad2Δ*::KANMX4 | *rad2Δ* | YDC-OB |
| 40 | *rad5Δ/ rad5Δ* | Isogenic to BY4743 *rad5Δ*::KANMX4 | *rad5Δ* | YDC-OB |
| 41 | *rad6Δ/ rad6Δ* | Isogenic to BY4743 *rad6Δ*::KANMX4 | *rad6Δ* | YDC-OB |
| 42 | *rad7Δ/ rad7Δ* | Isogenic to BY4743 *rad7Δ*::KANMX4 | *rad7Δ* | YDC-OB |
| 43 | *rad9Δ/ rad9Δ* | Isogenic to BY4743 *rad9Δ*::KANMX4 | *rad9Δ* | YDC-OB |
| 44 | *rad16Δ/ rad16Δ* | Isogenic to BY4743 *rad16Δ*::KANMX4 | *rad16Δ* | YDC-OB |
| 45 | *rad17Δ/ rad17Δ* | Isogenic to BY4743 *rad17Δ*::KANMX4 | *rad17Δ* | YDC-OB |
| 46 | *rad18Δ/ rad18Δ* | Isogenic to BY4743 *rad18Δ*::KANMX4 | *rad18Δ* | YDC-OB |
| 47 | *rad23Δ/ rad23Δ* | Isogenic to BY4743 *rad23Δ*::KANMX4 | *rad23Δ* | YDC-OB |
| 48 | *rad24Δ/ rad24Δ* | Isogenic to BY4743 *rad24Δ*::KANMX4 | *rad24Δ* | YDC-OB |
| 49 | *rad27Δ/ rad27Δ* | Isogenic to BY4743 *rad27Δ*::KANMX4 | *rad27Δ* | YDC-OB |
| 50 | *rad54Δ/ rad54Δ* | Isogenic to BY4743 *rad54Δ*::KANMX4 | *rad54Δ* | YDC-OB |
| 51 | *rad55Δ/ rad55Δ* | Isogenic to BY4743 *rad55Δ*::KANMX4 | *rad55Δ* | YDC-OB |
| 52 | *rad57Δ/ rad57Δ* | Isogenic to BY4743 *rad57Δ*::KANMX4 | *rad57Δ* | YDC-OB |
| 53 | *rad59Δ/ rad59Δ* | Isogenic to BY4743 rad59Δ::KANMX4 | *rad59Δ* | YDC-OB |
| 54 | *pso1Δ/pso1Δ* | Isogenic to BY4743 *pso1Δ*::KANMX4 | *pso1Δ* | YDC-OB |
| 55 | *ubc10Δ/ubc10Δ* | Isogenic to BY4743 *ubc10Δ*::KANMX4 | *ubc10Δ* | YDC-OB |
| 56 | *slx8Δ/slx8Δ* | Isogenic to BY4743 *slx8Δ*::KANMX4 | *slx8Δ* | YDC-OB |
| 57 | *bre1Δ/bre1Δ* | Isogenic to BY4743 *bre1Δ ::KANMX4* | *bre1Δ* | YDC-OB |
| 58 | *slx5Δ/slx5Δ* | Isogenic to BY4743 *slx5Δ*::KANMX4 | *slx5Δ* | YDC-OB |
| 59 | *hel2Δ/hel2Δ* | Isogenic to BY4743 *hel2Δ*::KANMX4 | *hel2Δ* | YDC-OB |
| 60 | *mms2Δ/mms2Δ* | Isogenic to BY4743 *mms2Δ*::KANMX4 | *mms2Δ* | YDC-OB |
| 61 | *def1Δ/def1Δ* | Isogenic to BY4743 *def1Δ*::KANMX4 | *def1Δ* | YDC-OB |
| 62 | *mms1Δ/mms1Δ* | Isogenic to BY4743 *mms1Δ*::KANMX4 | *mms1Δ* | YDC-OB |
| 63 | *crt10Δ/crt10 Δ* | Isogenic to BY4743 crt10Δ::KANMX4 | *crt10 Δ* | YDC-OB |
| 64 | *rad30Δ/rad30Δ* | Isogenic to BY4743 *rad30::KANMX4* | *rad30Δ* | YDC-OB |
| 65 | *rev1Δ/rev1Δ* | Isogenic to BY4743  *rev1Δ::KANMX4* | *rev1Δ* | YDC-OB |
| 66 | *ssa1Δ/ssa1Δ* | Isogenic to BY4743 *ssa1Δ::KANMX4* | *ssa1Δ* | YDC-OB |
| 67 | *ssa2Δ/ssa2Δ* | Isogenic to BY4743 *ssa2Δ::KANMX4* | *ssa2Δ* | YDC-OB |
| 68 | *ssa4Δ/ssa4Δ* | Isogenic to BY4743 *ssa4Δ::KANMX4* | *ssa4Δ* | YDC-OB |
| 69 | *ssb2Δ/ssb2Δ* | Isogenic to BY4743 ssb2Δ::KANMX4 | *ssb2Δ* | YDC-OB |
| 70 | *ssq1Δ/ssq1Δ* | Isogenic to BY4743 ssq1Δ::KANMX4 | *ssq1Δ* | YDC-OB |
| 71 | *lhs11Δ/lhs1Δ* | Isogenic to BY4743 *lhs1Δ::KANMX4* | *lhs1Δ* | YDC-OB |
| 72 | *ssb1Δ/ssb1Δ* | Isogenic to BY4743 *ssb1Δ::KANMX4* | *ssb1Δ* | YDC-OB |
| 73 | *hsp12Δ/ hsp12Δ* | Isogenic to BY4743 *hsp12Δ*::KANMX4 | *hsp12Δ* | YDC-OB |
| 74 | *hsp26Δ/ hsp26Δ* | Isogenic to BY4743 *hsp26Δ*::KANMX4 | *hsp26Δ* | YDC-OB |
| 75 | *hsp30Δ/ hsp30Δ* | Isogenic to BY4743 *hsp30Δ*::KANMX4 | *hsp30Δ* | YDC-OB |
| 76 | *hsp31Δ/ hsp31Δ* | Isogenic to BY4743 *hsp31Δ ::KANMX4* | *hsp31Δ* | YDC-OB |
| 77 | *hsp42Δ/ hsp42Δ* | Isogenic to BY4743 *hsp42Δ*::KANMX4 | *hsp42Δ* | YDC-OB |
| 78 | *hsp78Δ/ hsp78Δ* | Isogenic to BY4743 h*sp78Δ*::KANMX4 | *hsp78Δ* | YDC-OB |
| 79 | *hsp104Δ/ hsp104Δ* | Isogenic to BY4743 *hsp104*Δ::KANMX4 | *hsp104Δ* | YDC-OB |
| 80 | WT 4741 | MATa his3Δ1 leu2Δ met15Δ ura3Δ0 | WT | R. Nicholas Laribee |
| 81 | *tor1Δ* | BY4741; *tor1Δ::KanMX* | *tor1Δ* | R. Nicholas Laribee |
| 82 | *asf1Δ* | BY4741; *asf1Δ::KanMX* | *asf1Δ* | R. Nicholas Laribee |
| 83 | *vps75Δ* | BY4741; *vps75Δ::KanMX* | *vps75Δ* | R. Nicholas Laribee |
| 84 | *nap1Δ* | BY4741; *nap1Δ::KanMX* | *nap1Δ* | R. Nicholas Laribee |
| 85 | *sch9Δ* | BY4741; *sch9Δ::KanMX* | *sch9Δ* | R. Nicholas Laribee |
| 86 | *tco89Δ* | BY4741; *tco89Δ::KanMX* | *tco89Δ* | R. Nicholas Laribee |
| 87 | *asf1Δtor1Δ* | asf1Δtor1Δ | *asf1Δtor1Δ* | R. Nicholas Laribee |
| 88 | *asf1Δtor89Δ* | asf1Δtor89Δ | *asf1Δtco89Δ* | R. Nicholas Laribee |
