# Supplementary material for: Correction: Assessment of the Biological Pathways Targeted by Isocyanate Using N-Succinimidyl N-Methylcarbamate in Budding Yeast Saccharomyces cerevisiae
Source: PLoS One. 2024 Jul 5;19(7):e0306937. doi: 10.1371/journal.pone.0306937 (PMC11226043; doi:10.1371/journal.pone.0306937)

Raw data for revised version of figure 2A

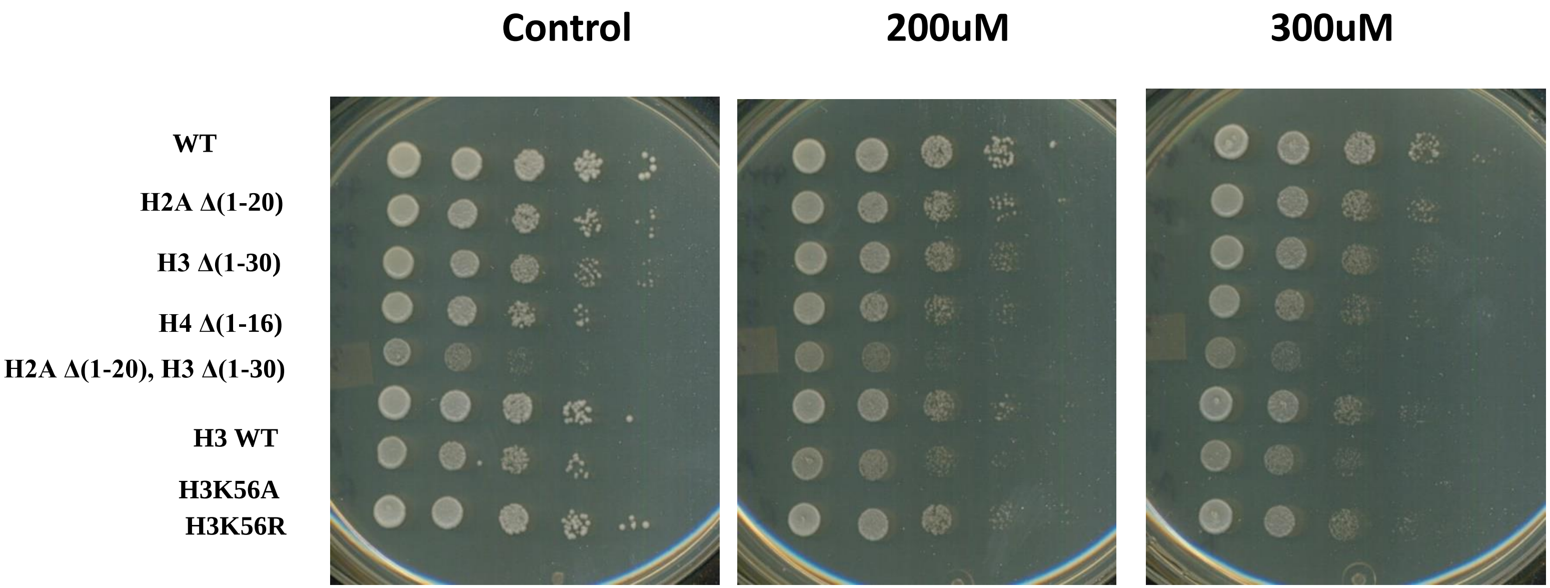

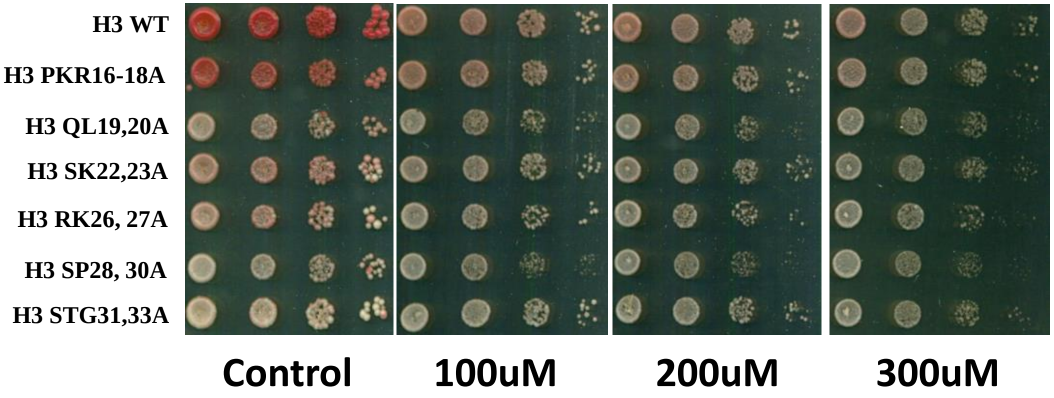

Raw data for revised version of figure 2B

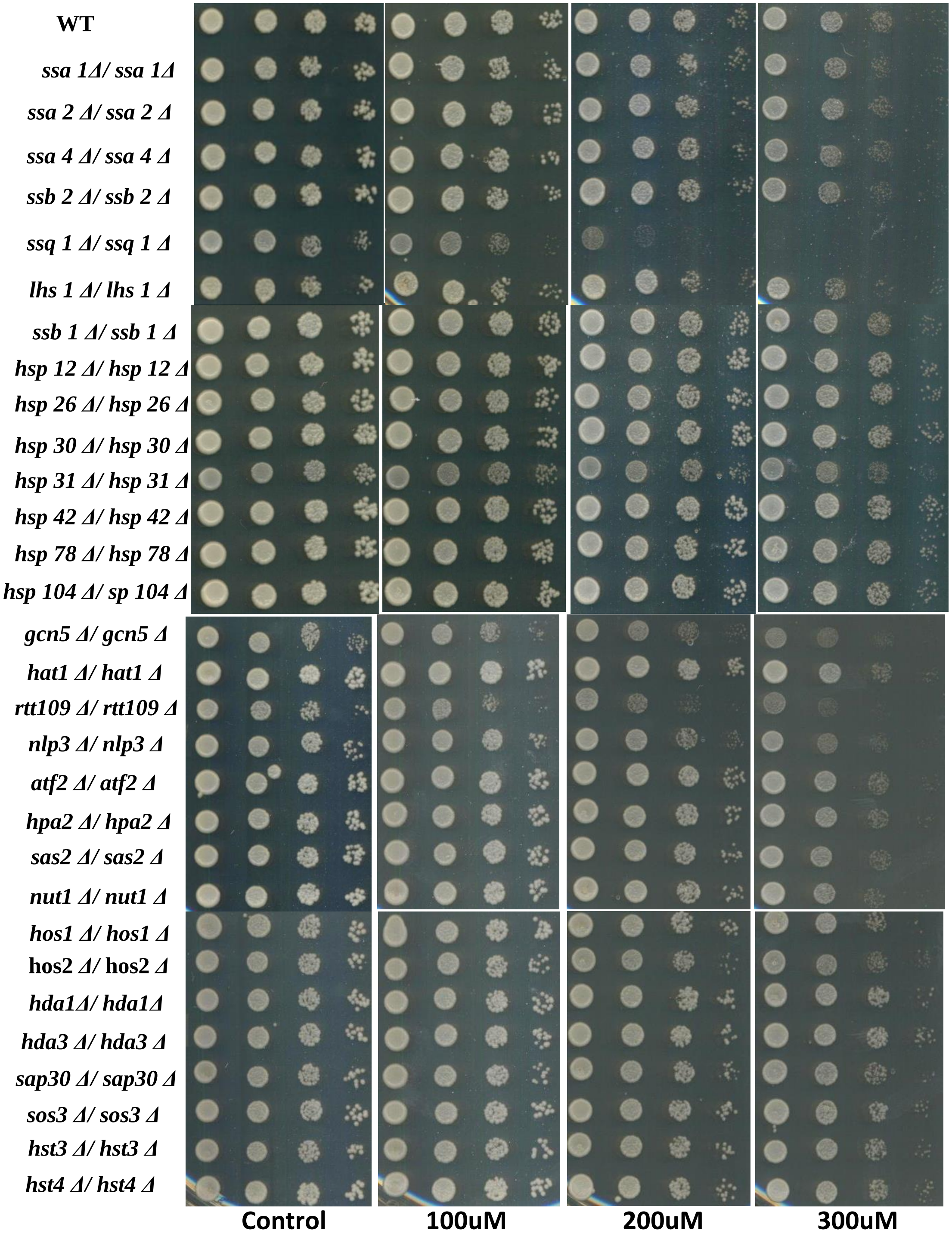

Raw data of revised version of figure 2C

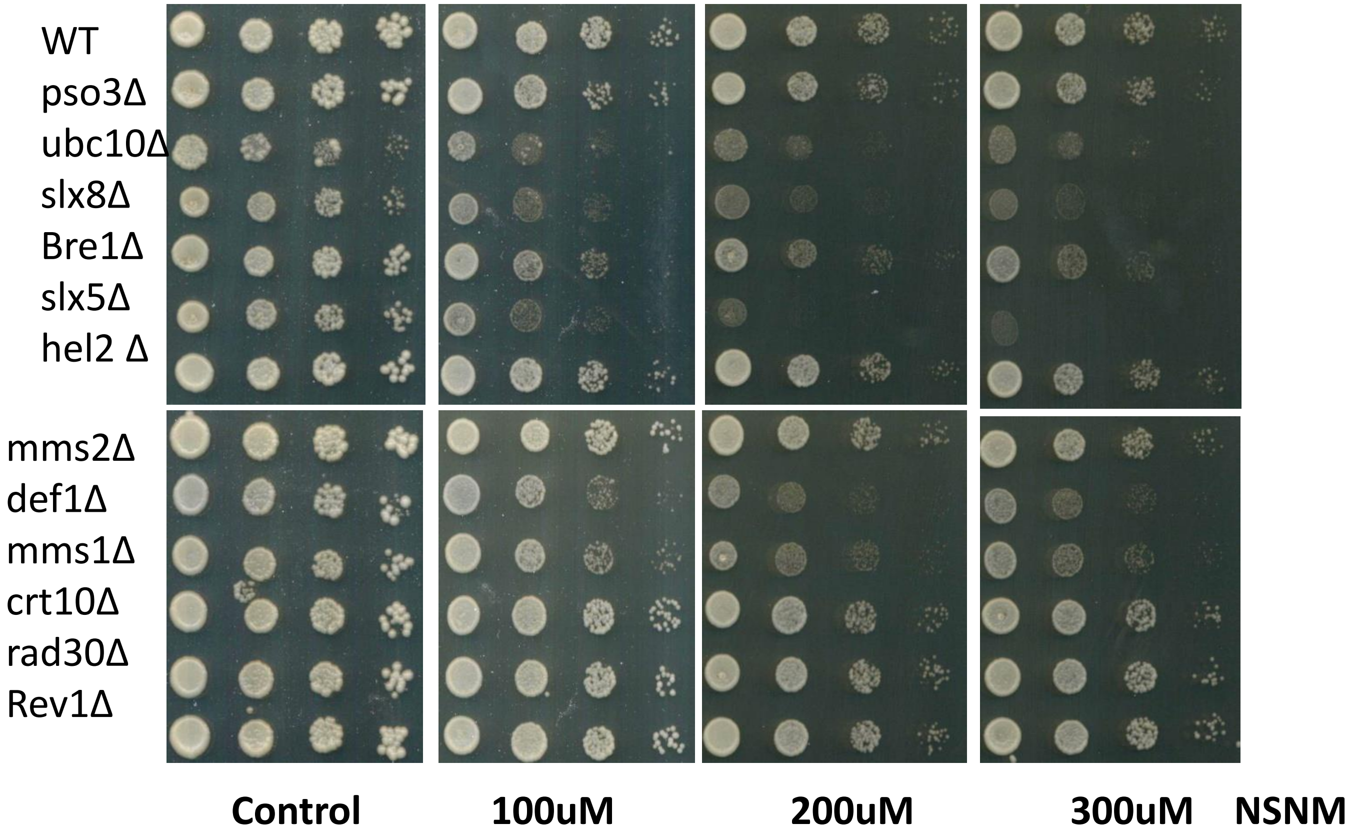

Raw data for revised version of figure 2D

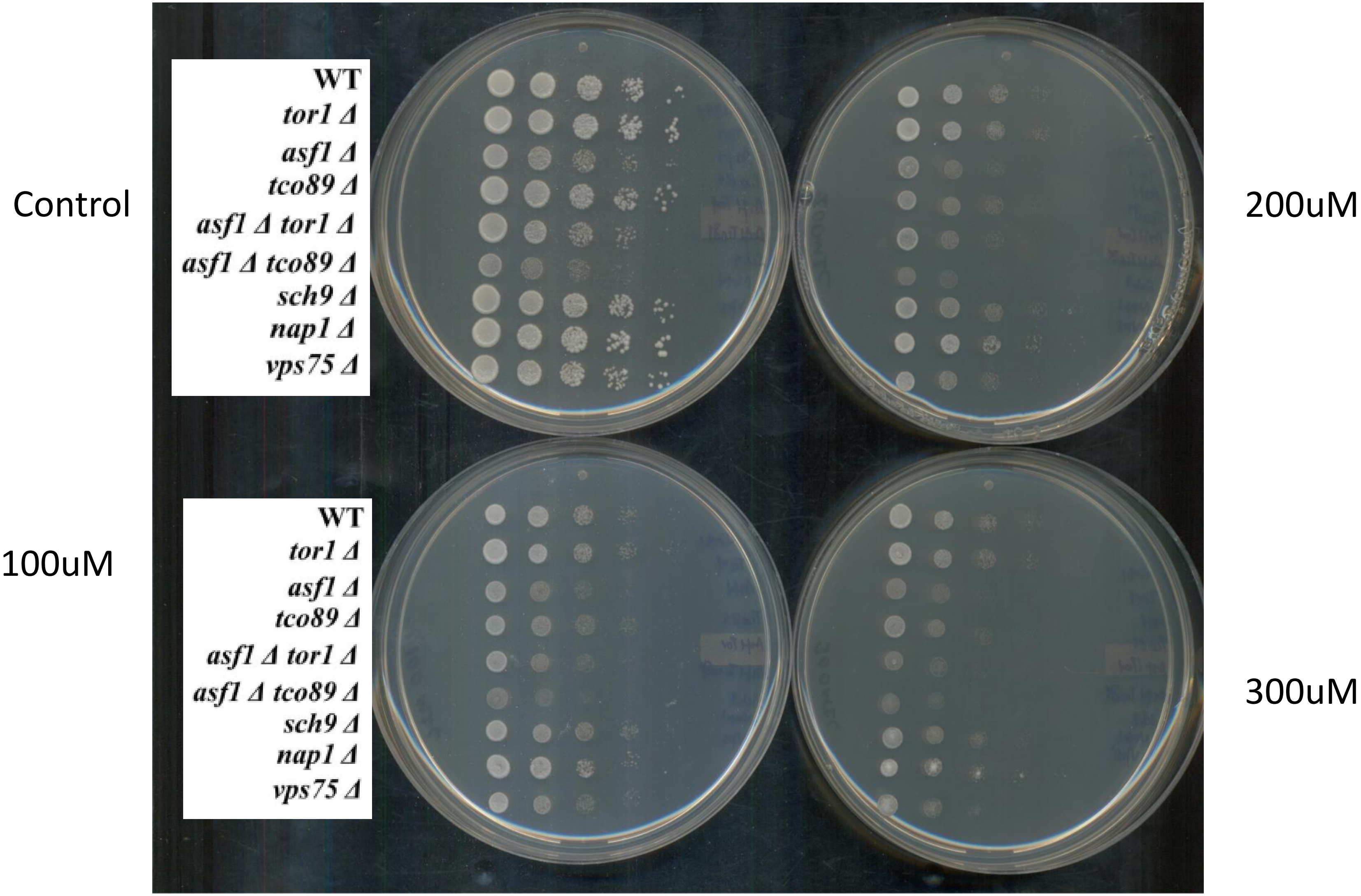

Raw data of figure 6B

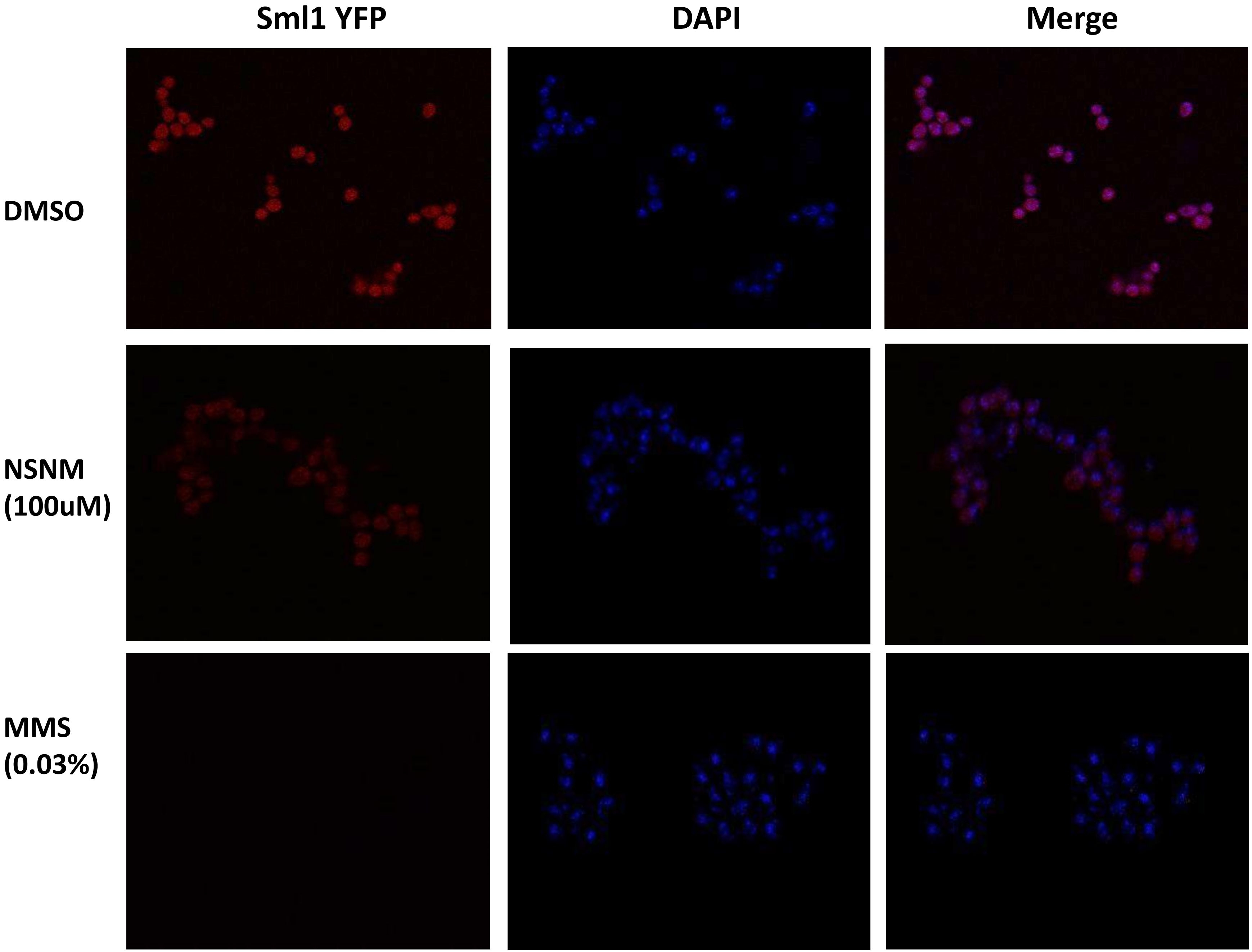

Raw data of figure 6C

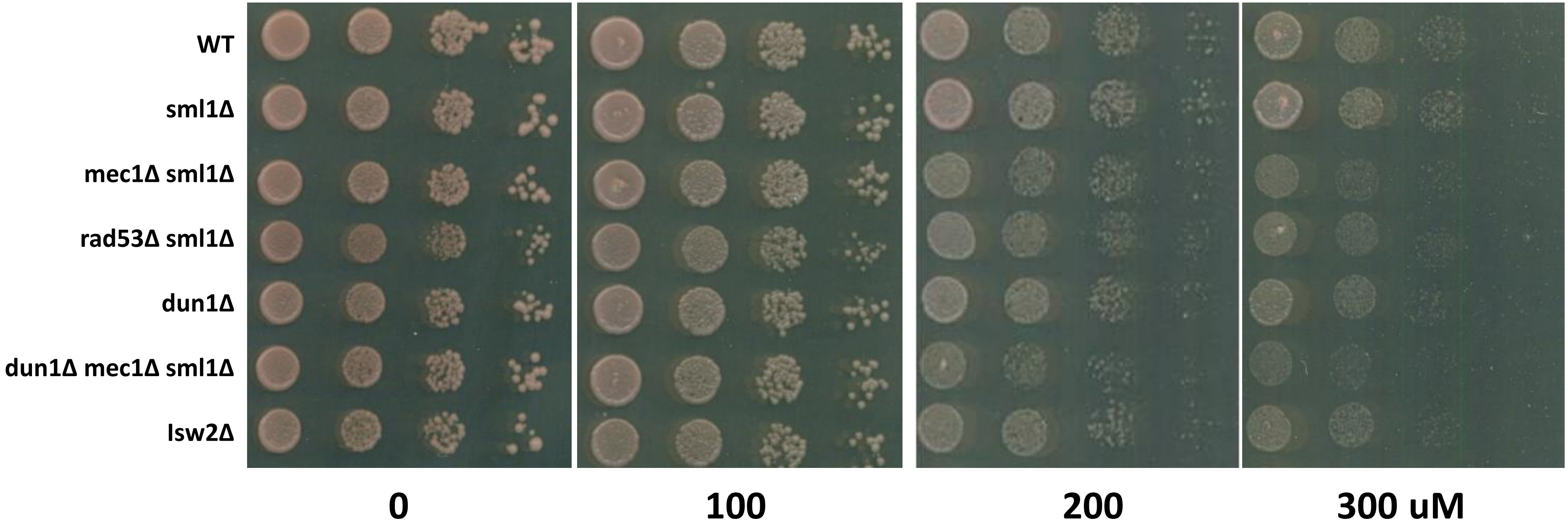

Raw data of figure 6D

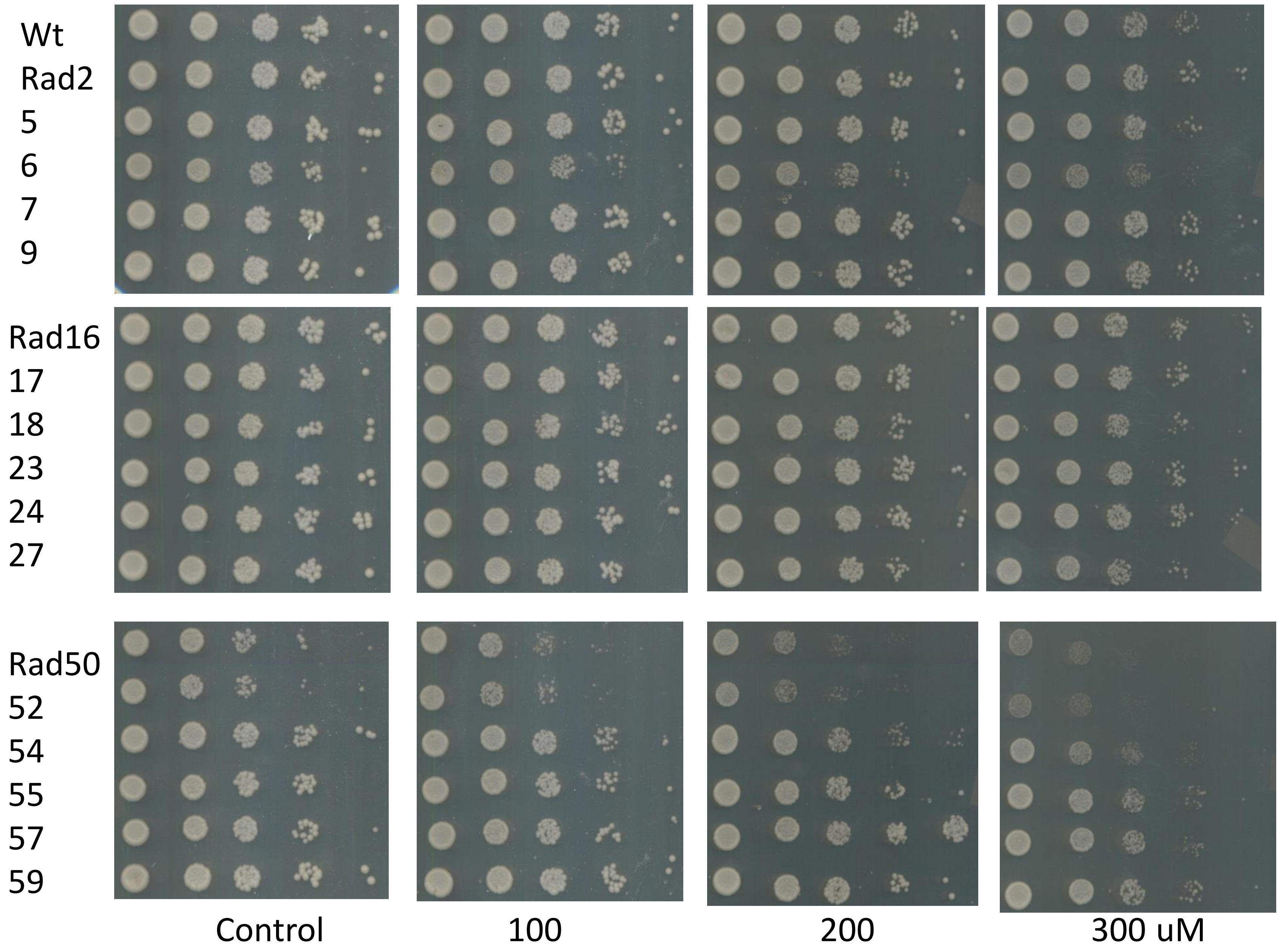

Raw data of figure 6E

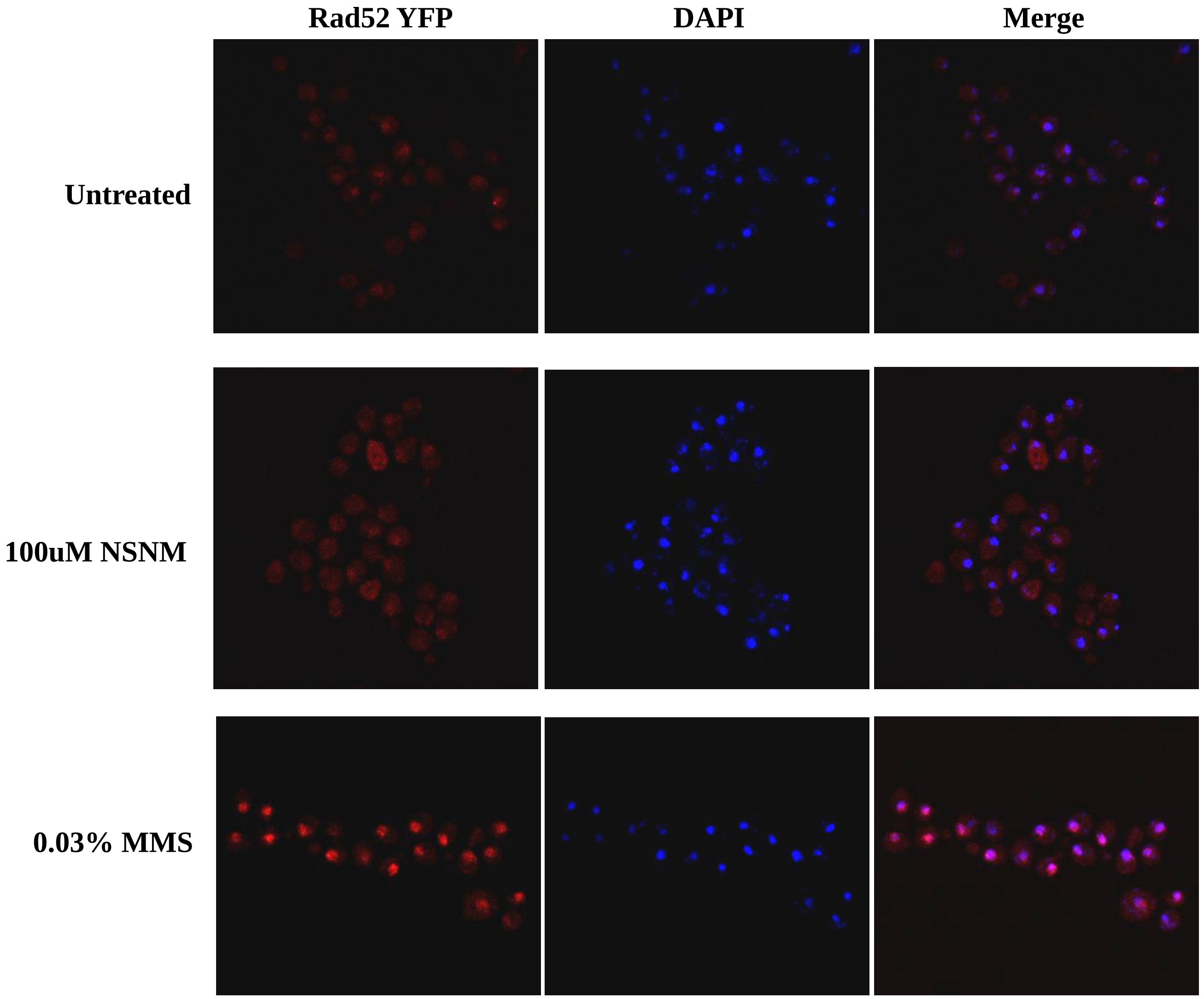

Supplement: S1 File — This file includes the uncropped images for the updated Fig 2 and for Fig 6 in [1]. (PDF) [file pone.0306937.s001.pdf]
